# Shrimp Lipid Droplet Protein Perilipin Involves in the Pathogenesis of AHPND-Causing *Vibrio parahaemolyticus*

**DOI:** 10.3390/ijms231810520

**Published:** 2022-09-10

**Authors:** Chuanqi Wang, Defu Yao, Mingming Zhao, Kaiyu Lu, Zhongyang Lin, Xiuli Chen, Yongzhen Zhao, Yueling Zhang

**Affiliations:** 1 Guangdong Provincial Key Laboratory of Marine Biotechnology, Institute of Marine Sciences, Shantou University, Shantou 515063, China; 2 STU-UMT Joint Shellfish Research Laboratory, Shantou University, Shantou 515063, China; 3 Guangxi Key Laboratory of Aquatic Genetic Breeding and Healthy Aquaculture, Guangxi Academy of Fishery Sciences, Nanning 530021, China

**Keywords:** shrimp, AHPND-causing *Vibrio parahaemolyticus*, perilipin, lipid droplets, ROS

## Abstract

Acute hepatopancreatic necrosis disease (AHPND), caused by a unique strain of *Vibrio parahaemolyticus* (*Vp* (AHPND)), has become the world’s most severe debilitating disease in cultured shrimp. Thus far, the pathogenesis of AHPND remains largely unknow. Herein, in *Litopenaeus vannamei*, we found that a *Vp* (AHPND) infection significantly increased the expression of lipid droplets (LDs) protein *Lv*Perilipin, as well as promoted the formation of LDs. In addition, the knockdown of *Lv*Perilipin increased the shrimp survival rate in response to the *Vp* (AHPND) infection, and inhibited the proliferation of *Vp* (AHPND). Furthermore, we demonstrated that *Lv*Perilipin depletion could increase the production of reactive oxygen species (ROS), which may be responsible for the decreased *Vp* (AHPND) proliferation. Taken together, our current data for the first time reveal that the shrimp lipid droplets protein Perilipin is involved in the pathogenesis of *Vp* (AHPND) via promoting LDs accumulation and decreasing ROS production.

## 1. Introduction

Acute hepatopancreatic necrosis disease (AHPND), a severe farmed debilitating disease known initially as early mortality syndrome (EMS), has been causing havoc in the shrimp aquaculture industry [1,2]. Since the first outbreak in China in 2009, AHPND has rapidly spread to many Southeast Asian and American countries, including Vietnam (2010), Malaysia (2011), Thailand (2012), Mexico (2013), the Philippines (2015), and South America (2016), resulting in massive mortality of shrimp and enormous economic losses [1,3,4]. AHPND is caused by a unique strain of *Vibrio parahaemolyticus* (termed here as *Vp* (AHPND)). It contains a 70 kbp plasmid (pVA1) that encodes the deadly binary PirA and PirB pore-forming toxins [5,6]. PirA/B toxins mainly target and damage the shrimp’s stomach, hepatopancreas, and gut, resulting in cell dysfunction and mass mortality (up to 100%) [7,8,9]. PirA is found to mainly play an auxiliary role in the pathogenesis of *Vp* (AHPND), while PirB alone could result in cellular damage and exhibit typical symptoms of AHPND [5,10]. PirB interacts with and dephosphorylates histone H3, resulting in hemocytes apoptosis [11]. A *Vp* (AHPND) infection also facilitated Rho-signaling pathway activation, which plays a critical role in the disintegration of the shrimp’s stomach epithelial cells. This might allow the formation of intercellular gaps and help PirA/B toxins and *Vp* (AHPND) reach the hepatopancreas from the stomach for infection [7,12]. Moreover, a recent report showed that *Vp* (AHPND) interfered with lipid metabolism and primary bile acid synthesis in shrimp using a metabolomics study [13]. These studies indicated that *Vp* (AHPND) adopts various strategies to survive in the shrimp. Even though many efforts have been made, the pathogenesis of *Vp* (AHPND) remains poorly understood and needs to be further investigated.

Perilipin protein family (Perilipin 1–5) are the most abundant proteins associated with lipid droplets (LDs) [14]. Perilipin is located at the surface of intracellular LDs and acts as the critical regulator of cellular lipid metabolism, directly controlling the formation or degradation of intracellular LDs [15]. Perilipin 1 could promote unilocular lipid droplet formation in adipocytes by activating the fat-specific protein of 27 (Fsp27) [16]. Furthermore, Perilipin 1 could interact with comparative gene identification-58 (CGI-58) and adipose triglyceride lipase (ATGL) to initiate triglyceride hydrolysis [17]. After the knockdown of Perilipin 2, intracellular LDs are significantly reduced in number and diameter [18,19], while the AMPK-dependent phosphorylation of Perilipin 2 triggers its degradation by chaperone-mediated autophagy (CMA), thus promoting the recruitment of autophagy effector proteins and cytosolic lipases to degrade the lipid droplets [20,21]. Perilipin 3 could differentially regulate skeletal muscle lipid oxidation and intracellular lipid degradation [22,23]. In HepG2 cells, the overexpression of Perilipin 5 facilitated LDs formation and reduced the cellular reactive oxygen species (ROS) level. Interfering Perilipin 5, meanwhile, showed the opposite effects [24]. Interestingly, previous research had found that Perilipin accumulation and LDs formation occurred during cell infection with various pathogens, including intracellular viruses, bacteria, and parasites. LDs function as platforms for viral assembly and sources of nutrients for intracellular bacteria and parasites [25,26]. Surprisingly, a recent study revealed that LDs are innate immune hubs, integrating cell metabolism and immune response to pathogen invasion. During lipopolysaccharide (LPS) stimulation, Perilipin 2 was significantly up-regulated and multiple immune proteins, including viperin, IGTP, IIGP1, TGTP1, IFI47, and cathelicidin, were nucleated around Perilipin 2 to form an anti-pathogenic complex [27].

In the present study, we found that the *Litopenaeus vannamei* Perilipin (*Lv*Perilipin) was up-regulated during *Vp* (AHPND) infection. Furthermore, the increased expression of *Lv*Perilipin could promote the biogenesis of lipid droplets and suppress ROS production, which facilitates the *Vp* (AHPND) proliferation. The current findings provide novel insight into the pathogenesis of *Vp* (AHPND), which would contribute to *Vp* (AHPND) control in shrimp aquaculture.

## 2. Results

### 2.1. Sequence and Phylogenetic Analysis of LvPerilipin

**Figure 1:**
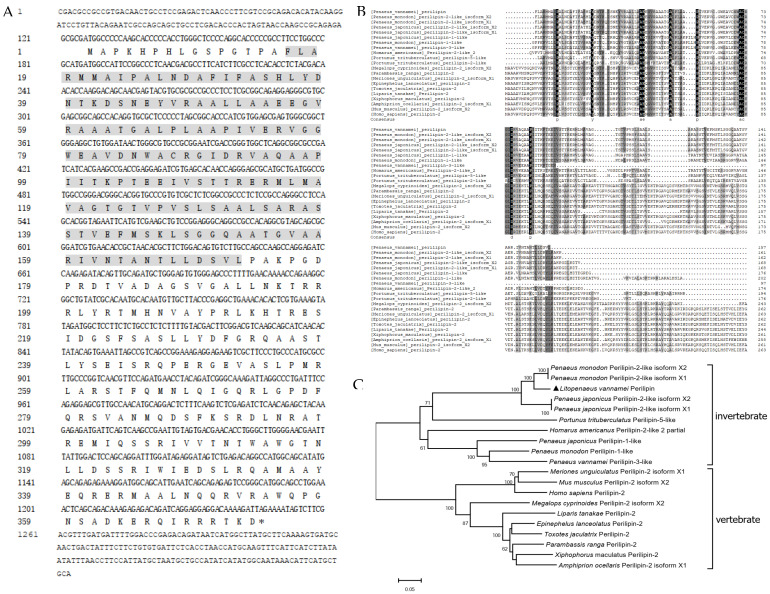
Sequence analysis of *Lv*Perilipin. (**A**) Nucleotide and deduced amino acid sequence of *Lv*Perilipin. The Perilipin characteristic domain (residues 16-172) was indicated with a grey shadow. (**B**) Multiple sequence alignment among Perilipin of *L. vannamei* and other species. (**C**) Phylogenetic analysis based on the amino acid sequences of *Lv*Perilipin. Protein sequences of different species were listed below: *L. vannamei* Perilipin (ON745208), *Penaeus monodon* Perilipin-2-like isoform X2 (XP_037803900.1), *P. monodon* Perilipin-2-like isoform X1 (XP_037803899.1), *Penaeus japonicus* Perilipin-2-like isoform X2 (XP_042858379.1), *P. japonicus* Perilipin-2-like isoform X1 (XP_042858378.1), *P. japonicus* Perilipin-1-like (XP_042878935.1), *P. monodon* Perilipin-1-like (XP_037804123.1), *P. vannamei* Perilipin-3-like (XP_027222535.1), *Homarus americanus* Perilipin-2-like 2, partial (KAG7166848.1), *Portunus trituberculatus* Perilipin-5-like (XP_045104753.1), *P. trituberculatus* Perilipin-2-like (XP_045102117.1), *Megalops cyprinoides* Perilipin-2 isoform X2 (XP_036407853.1), *Parambassis ranga* Perilipin-2 (XP_028285006.1), *Meriones unguiculatus* Perilipin-2 isoform X1 (XP_021505457.1), *Epinephelus lanceolatus* Perilipin-2 (XP_033465614.1), *Toxotes jaculatrix* Perilipin-2 (XP_040887796.1), *Liparis tanakae* Perilipin-2 (TNN83914.1), *Xiphophorus maculatus* Perilipin-2 (XP_014324455.1), *Amphiprion ocellaris* Perilipin-2 isoform X1 (XP_023150912.1), *Mus musculus* Perilipin-2 isoform X2 (XP_011248209.1), *Homo sapiens* Perilipin-2 (NP_001113.2).

The full-length of *Lv*Perilipin was obtained from the *L. vannamei* transcriptome analyzed in our laboratory (unpublished). The *Lv*Perilipin gene (GenBank accession No. ON745208) was 1440 bp in length, which included a 1121 bp open reading frame (ORF) encoding a 373-amino acid protein (molecular mass: 40.802 kDa) (Figure 1A). InterPro analysis displayed that *Lv*Perilipin contained a Perilipin characteristic domain (16–172 aa) (Figure 1A). A multiple sequences alignment suggested that Perilipin characteristic domains were not conserved in vertebrates and invertebrates (Figure 1B). A phylogenetic analysis revealed that Perilipins in vertebrates and invertebrates tended to form separate clusters. Evolutionarily, *Lv*Perilipin was associated with the Perilipins of crustaceans, such as *Penaeus monodon* and *Portunus trituberculatus* (Figure 1C).

### 2.2. Vp (AHPND) Infection Up-Regulates LvPerilipin Expression and Increases LDs Biogenesis in Shrimp Hemocytes

**Figure 2:**
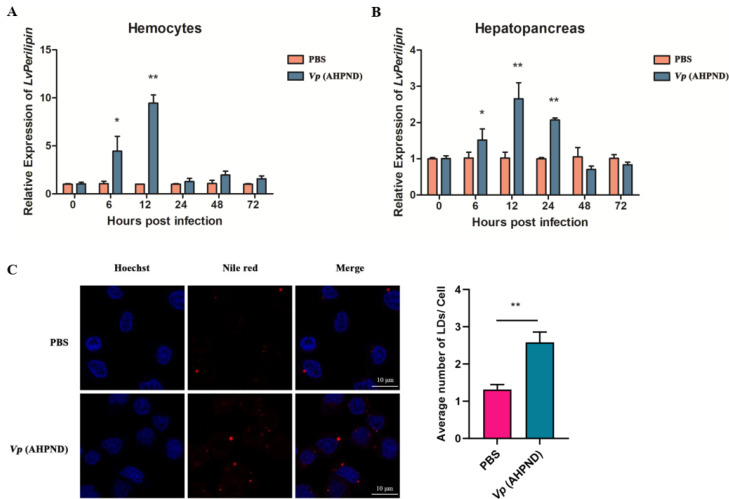
*Vp* (AHPND) infection increased *Lv*Perilipin expression and promoted LDs biogenesis. Expression of *Lv*Perilipin in shrimp hemocytes (**A**) and hepatopancreas (**B**) after *Vp* (AHPND) infection, as determined using qRT-PCR. Hemocytes and hepatopancreas were collected at 0, 6, 12, 24, 48, and 72 h post-*Vp* (AHPND) injection. The total RNA of each sample was extracted and synthesized for the first strand of cDNA. *LvEF-1α* was used as the internal control. (**C**) Results of LDs staining using Nile red were observed from hemocytes of shrimps. The hemocytes were collected at 12 h after PBS (negative control) or *Vp* (AHPND) injection, subjected to LDs marking, and observed under a laser scanning confocal microscope. The average number of LDs per cell was quantified from ~100 cells. The figure is representative of three independent experiments. Statistical significance was set at * *p* < 0.05, ** *p* < 0.01. Scale bars = 10 μm.

To investigate the relationship between *Lv*Perilipin and *Vp* (AHPND) infection, we performed qRT-PCR to detect the expression of an *Lv*Perilipin flowing *Vp* (AHPND) infection. The results showed that *Lv*Perilipin was significantly up-regulated approximately five-fold at 6 hpi and nine-fold at 12 hpi in hemocytes (Figure 2A). While in hepatopancreas, the expression of *Lv*Perilipin was up-regulated about 1.5-fold at 6 hpi, 2.5-fold at 12 hpi, and 2-fold at 24 hpi (Figure 2B). These results indicated that *Vp* (AHPND) positively regulates the expression of *Lv*Perilipin. Various pathogens could promote LDs formation [25,26], so we then investigated the effect of *Vp* (AHPND) on LDs biogenesis. Healthy shrimp were injected with PBS (as a control) or *Vp* (AHPND). Twelve hours later, the hemocytes were collected, subjected to LDs staining, and observed by a laser scanning confocal microscope. The results showed that the number of LDs significantly increased more than two-fold after *Vp* (AHPND) infection (Figure 2C). These cumulative evidences suggest that *Vp* (AHPND) infection up-regulates the expression of *Lv*Perilipin and increases LDs biogenesis in shrimp hemocytes.

### 2.3. LvPerilipin Promotes Vp (AHPND) Infection and Shrimp Mortality

**Figure 3:**
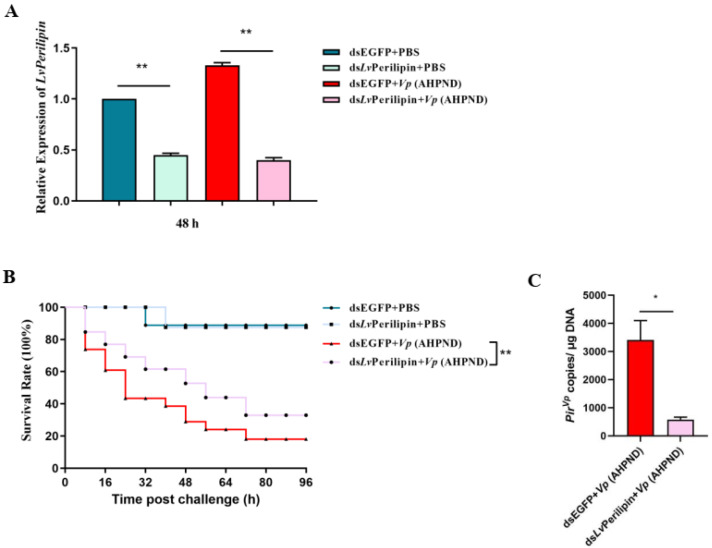
The effect of *Lv*Perilipin on shrimp survival rate and *Vp* (AHPND) proliferation. (**A**) The expression level of *Lv*Perilipin after RNAi. RNAi assay was performed as described in 4.4. The shrimp hemocytes were collected at 48 h post-*Vp* (AHPND) infection and subjected to determine the *Lv*Perilipin expression using qRT-PCR. (**B**) Silencing of *Lv*Perilipin contributed to shrimp survival during *Vp* (AHPND) infection. The survival rate of shrimps (*n* = 40 per group) was determined after intramuscular injection with dsEGFP (negative control) or ds*Lv*Perilipin followed by *Vp* (AHPND) or PBS (negative control) injection, and the dead shrimp of each group was recorded at 8 h intervals. The shrimp survival rate was calculated using the product-limit method of Kaplan-Meier, and the significance was compared using the log-rank test. (**C**) Knockdown of *Lv*Perilipin suppressed *Vp* (AHPND) proliferation. DsEGFP (control group) and ds*Lv*Perilipin were injected into shrimps. After 12 h, the two groups were injected with 1 × 10^5^ unit *Vp* (AHPND). The shrimp hemolymph (*n* = 3 per group) was collected to extract genomic DNA, and 100 ng of each DNA was subjected to qRT-PCR analysis. Pir*^vp^* copies in 1 μg of shrimp genomic DNA were then calculated. The data were statistically analyzed via the student’s *t*-test (* *p* < 0.05, ** *p* < 0.01).

To figure out the effect of *Lv*Perilipin on *Vp* (AHPND) proliferation and shrimp mortality, we employed RNAi to knockdown the expression level of *Lv*Perilipin first. Healthy shrimp were intramuscularly injected with dsEGFP (as a control) or ds*Lv*Perilipin and were divided into four groups. Two groups (dsEGFP and ds*Lv*Perilipin injection) were injected with PBS, and the rest were challenged with *Vp* (AHPND) subsequently. The silencing efficiency of *Lv*Perilipin was assessed by qRT-PCR at 48 h post-*Vp* (AHPND) injection. The results showed that the transcriptional level of *Lv*Perilipin was suppressed in the hemocytes injected with ds*Lv*Perilipin as compared to the controls (Figure 3A). After the injection of shrimps with *Vp* (AHPND), the number of dead shrimps was recorded every eight hours. As shown in Figure 3B, compared with the dsEGFP control group, the survival rate of the shrimps was significantly increased in the ds*Lv*Perilipin-treated group. In addition, the number of *Vp* (AHPND) in the hemolymphs significantly decreased after *Lv*Perilipin silencing (Figure 3C). These results suggested that *Lv*Peirlipin plays a negative role in the proliferation of *Vp* (AHPND) during infection.

### 2.4. LvPerilipin Is Located on the Surface of LDs and Promotes LDs Biogenesis

**Figure 4:**
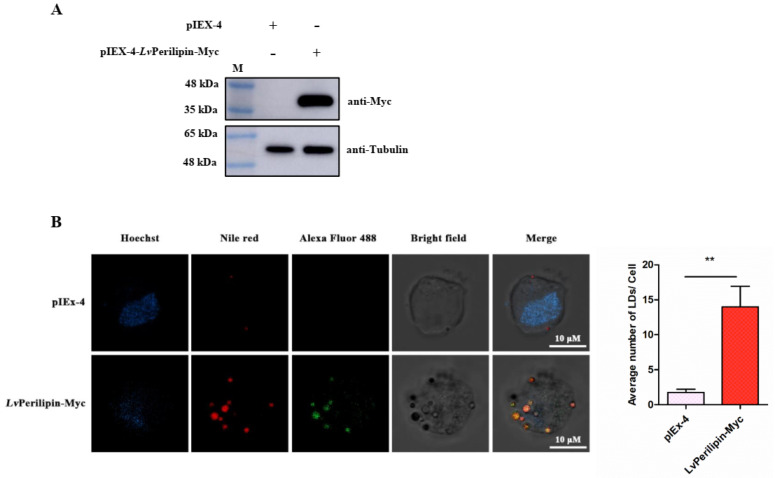
*Lv*Perilipin facilitates LDs biogenesis. (**A**) Western blotting analysis indicated that Myc-tagged *Lv*Perilipin was successfully expressed in High Five cells. (**B**) Immunofluorescence analysis revealed that *Lv*Perilipin colocalized with LDs and promoted LDs biogenesis. High Five cells were transfected with Myc-*Lv*Perilipin. After transfection for 36 h, immunofluorescence analysis was performed with primary mouse anti-Myc and Alexa Fluor 488 anti-mouse antibodies. The cells were then stained with Nile red to mark LDs. Finally, a confocal laser-scanning microscope was used to observe the cells. The number of LDs puncta in each cell was counted. Statistical significance was set at ** *p* < 0.01. Scale bars = 10 μm.

In vertebrates, the numbers of the Perilipin family play an important role in LDs formation and degradation [15]. Therefore, we overexpressed *Lv*Perilipin in High Five cells to determine the effect of *Lv*Perilipin on LDs biogenesis. After 36 h post-transfection, 10^5^ cells stuck to the confocal Petri dishes were used for immunofluorescence analysis, and the rest were subjected to Western blotting analysis. The results showed that the Myc-tagged *Lv*Perilipin band could be detected using the anti-Myc antibody at the predicted molecular weight between 35 kDa and 48 kDa, indicating that the Myc-tagged *Lv*Perilipin successfully expressed in the High Five cells (Figure 4A). The immunofluorescence analysis revealed a strong colocalization of *Lv*Perilipin with LDs (Figure 4B). Moreover, the number of LDs significantly increased in *Lv*Perilipin overexpressed cells (Figure 4B), which suggests that *Lv*Perilipin could promote LDs biogenesis.

### 2.5. LvPerilipin Inhibits ROS Production While Vp (AHPND) Infection

**Figure 5:**
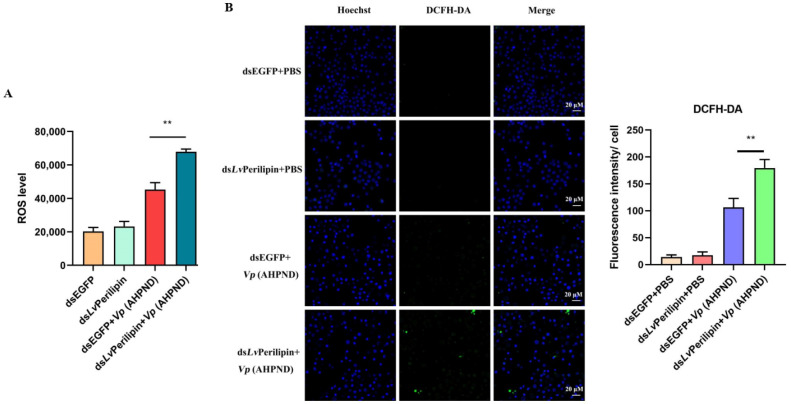
The effect of *Lv*Perilipin on the production of ROS in shrimp hemocytes upon *Vp* (AHPND) infection. (**A**) DsEGFP (control group) and ds*Lv*Perilipin (experimental group) were injected into shrimp. The two groups were then injected with PBS (negative control) or 1 × 10^5^ unit *Vp* (AHPND). After 12 h post-*Vp* (AHPND) injection, the shrimp hemocytes were labeled by DCFH-DA. The samples were detected using a microplate reader at 488 nm excitation wavelength and 525 nm emission wavelength. (**B**) The ROS levels of shrimp hemocytes were detected using a confocal laser-scanning microscope. Representative images are displayed. Scale bar = 20 μm. Statistical significance was set at ** *p* < 0.01.

According to the previous report, Perilipin 5 was responsible for the contact of LDs and mitochondria, up-regulated mitochondrial function-related genes, and reduced cellular ROS levels [24]. The ROS generated during the infection can kill the bacteria directly and perform critical regulatory functions in the innate immune system [28,29]. Therefore, we explored further to determine the effect of *Lv*Perilipin on ROS production. The shrimps were intramuscularly injected with either dsEGFP (control group) or ds*Lv*Perilipin (experimental group), which was followed by an injection with PBS or Vp (AHPND), respectively. After 12 h, shrimp hemocytes were collected and incubated with 10 μM of a DCFH-DA fluorescence probe. Finally, ROS levels were detected by the microplate reader or the confocal laser-scanning microscope. The results showed that the knockdown of *Lv*Perilipin could significantly increase ROS during *Vp* (AHPND) infection (Figure 5A,B). Thus, ROS production was suppressed by increasing *Lv*Perilipin during *Vp* (AHPND) infection.

## 3. Discussion

Numerous pathogens are able to modulate the host lipid metabolism. An obvious characterization of intracellular pathogens invasion is an accumulation of LDs in infected cells [25,30]. For instance, bacterial components, such as lipopolysaccharide (LPS) and lipoarabinomannan (LAM) [31,32], could trigger LDs formation in macrophages. *Burkholderia pseudomallei* induced the LDs formation in A549 cells via NR1D2-mediated PNPLA2/ATGL suppression to block autophagy [33]. A *Mycobacterium bovis* bacillus Calmette–Guérin (BCG) infection led to the PPARγ activation and LDs formation [34]. Additionally, multiple viruses, such as SARS-CoV-2 were seen to modulate lipids synthesis and uptake pathways to promote the biogenesis of LDs, which benefit virus proliferation in different human cell lines [35]. In shrimp, a metabolomics study found that AHPND-causing *V. parahaemolyticus* interfered with lipid metabolism and primary bile acid synthesis [12]. Therefore, *Vp* (AHPND) infection might cause an anomalous change in LDs. In this study, the Nile red staining results showed that LDs exist in the cytoplasm of shrimp hemocytes with or without bacterial infection, and the extracellular bacteria *Vp* (AHPND) led to LDs accumulation significantly (Figure 2C), suggesting the involvement of critical factors during *Vp* (AHPND) infection.

Perilipin functions as the major surface protein of LDs in non-adipose tissues and plays an essential role in LDs formation, incorporation, and accumulation [14,36,37]. However, the role of Perilipin in invertebrates during pathogenic infection is largely unknown. In the present study, the shrimp Perilipin (*Lv*Perilipin) was firstly cloned and characterized from *L. vannamei*, and only one homolog of Perilipin was found in the shrimp transcriptome and genome database. An amino acids sequence analysis found that *Lv*Perilipin contained a Perilipin characteristic domain (16–172 aa) (Figure 1A). Furthermore, immunofluorescence analysis showed that *Lv*Perilipin colocalized with LDs in High Five cells (Figure 4B), suggesting that *Lv*Perilipin is a member of the constitutive proteins of LDs. During *Vp* (AHPND) infection, *Lv*Perilipin expression was markedly increased in shrimp hemocytes and hepatopancreas (Figure 2A,B). Considering the role of *Lv*Perilipin in LDs formation, we inferred that the increased expression of *Lv*Perilipin promotes the formation of LDs during infection. The overexpression analysis in High Five cells suggested that *Lv*Perilipin was responsible for LDs formation (Figure 4B).

Excess fatty acids are acquainted with being “lipid toxic” inside the cells. Some studies have highlighted that cells protect themselves from “toxic” 1,2-DAG by oxidizing fatty acids or storing TAGs in LDs [38,39]. Furthermore, LDs also act as innate immune hubs after bacteria infection, and immune proteins of LDs, such as viperin and histone H2A, are able to inhibit the proliferation of viruses and bacteria [26,40,41]. However, in this work, we found that the knockdown of *Lv*Perilipin, referred to as the inhibition of LDs accumulation, suppressed *Vp* (AHPND) proliferation and improved shrimp survival (Figure 3). It is noted that *Vp* (AHPND) is an extracellular bacteria, which can not utilize the nutrition of intracellular LDs for proliferation. Therefore, we considered that the phenomenon of LDs accumulation indicated that shrimp lipid metabolism interfered during *Vp* (AHPND) infection. As reported, Perilipin was responsible for the contact of LDs and mitochondria, up-regulated mitochondrial function-related genes, and reduced cellular ROS levels [23]. We found that the knockdown of *Lv*Perilipin significantly increased ROS levels (Figure 5). It suggested that the increase in *Lv*Perilipin expression inhibited ROS production and weakened the host immune response during *Vp* (AHPND) infection.

In conclusion, we reported that shrimp Perilipin (*Lv*Perilipin) is responsible for LDs formation and is involved in the pathogenesis of *Vp* (AHPND) via inhibiting host ROS production. The work highlights novel therapeutic targets for controlling *Vp* (AHPND) infection.

## 4. Materials and Methods

### 4.1. Shrimp Culture and Bacteria Challenge

Healthy adult shrimp (*L. vannamei*), 10–15 g approximately, were purchased from Huaxun Aquatic Product Corporation (Shantou, Guangdong, China). Before processing, the shrimp were kept in air-pumped circulating seawater for three days and were detected to be free of *Vp* (AHPND) by PCR diagnosis. *Vp* (AHPND), stored in our laboratory, were cultured in a Tryptone Soya Broth (TSB) medium (tryptone: 15 g/L, soytone: 5 g/L, NaCl: 5 g/L, pH 7.2) at 37 °C with shaking and were diluted in sterile PBS (140 mM NaCl, 3 mM KCl, 8 mM Na_2_HPO_4_, 1.5 mM KH_2_PO_4_, pH 7.4). After the period of laboratory acclimatization, 40 healthy shrimps were injected with 100 μL *Vp* (AHPND) (1 × 10^5^ CFU/mL). Shrimps injected with 100 μL PBS were set as negative controls.

### 4.2. Total RNA Extraction, cDNA Synthesis, and Genomic DNA Extraction

TRIzol reagent (Molecular Research Center, Inc., Cincinnati, OH, USA) was used for total RNA isolation from shrimp hemocytes or hepatopancreas. Without removing residual genomic DNA, the first strand of cDNA was synthesized by TransScript One-Step gDNA Removal and cDNA Synthesis SuperMix (TransGen Biotech, Beijing, China) according to the manufacturer’s instructions. For the number of *Vp* (AHPND) detections, genomic DNA from the shrimp hemolymphs was extracted using the TIANGEN Marine Animal DNA Kit (TIANGEN, Beijing, China) according to the manufacturer’s instructions.

### 4.3. Sequence and Phylogenetic Analysis

The characteristic domains of *Lv*Perilipin were predicted using the InterPro online program (https://www.ebi.ac.uk/interpro/, accessed on 2 December 2021). The sequence of *Lv*Perilipin and its homologs from other species were derived from the National Center For Biotechnology Information (NCBI) databases. MEGA7 software (Version 7.0, Philadelphia, PA, USA) was used to construct the phylogenetic tree using the Neighbor-Joining (NJ) method based on the full-length amino acid sequence of Perilipin proteins.

### 4.4. RNAi Assay

RNA interference (RNAi) was used to knock down the expression of *Lv*Perilipin to determine its role in *Vp* (AHPND) infection. The synthesis of dsEGFP (control) and ds*Lv*Perilipin, with the primers listed in Table S1, was proceeded using a HiScribeTM T7 Quick High Yield RNA Synthesis Kit (New England Biolabs, CA, USA) according to the manufacturer’s instructions. After determining the concentration, 100 μL of PBS containing 10 μg of dsEGFP or ds*Lv*Perilipin was intramuscularly injected into shrimps twice at a 24 h interval. After that, the shrimps were injected with *Vp* (AHPND) (1 × 10^5^ CFU/mL) or PBS 12 h later (*n* = 50). In order to determine RNAi efficiency, shrimp hemocytes (*n* = 3 per group, 48 hpi) were collected and used for RT-qPCR analysis. The dead shrimp of each group was monitored every eight hours until four days post-*Vp* (AHPND) infection. The log-rank test method (GraphPad Prism software, Version 9.2.0, GraphPad Software, San Diego, CA, USA) was used to analyze the differences between groups.

### 4.5. qRT-PCR

The shrimp hemocytes and hepatopancreas samples were collected at 0, 6, 12, 24, and 48 h after injection with *Vp* (AHPND) or PBS (control group). After RNA extraction and cDNA synthesis, qRT-PCR was performed using the RealStar Green Power Mixture (GenStar, Beijing, China) in a LightCycler^®^ 480 II Real-Time PCR system (Roche, Basel, Switzerland). The RT-qPCR program was one cycle of pre-denaturation for 10 min at 95 °C, 40 cycles of 95 °C for 15 s, and 60 °C for 30 s. The primers of *Lv*Perilipin and *Lv*EF-1α (internal control) are listed in Table S1. The relative mRNA level of *Lv*Perilipin was determined via the 2^.^^−ΔΔCt^ method. For the shrimp hemolymph samples (*n* = 3 per group), after quantifying the extracted total DNA, 100 ng of each DNA was subjected to RT-qPCR with the primer set PirB-F/PirB-R (Table S1) as described by Lai et al. [10]. Pir*^vp^* copies in 1 μg of shrimp genomic DNA were then calculated. Statistical significance was set at * *p* < 0.05, ** *p* < 0.01.

### 4.6. Plasmid Construction and Cell Transfection

The ORF of *L. vannamei* Perilipin (*Lv*Perilipin), fused with the FLAG tag at the Myc-tag at the N-terminus, was cut by BamH I and Hind III (TaKaRa Bio Inc., Dalian, China) and cloned into a pIEX-4 vector (Novagen, Madison, WI, USA) to express the Myc-tagged fusion protein in eukaryotic cells. The primers used for plasmid construction are listed in Table S1. Before DNA transfection, High Five cells were seeded into a six-well culture plate and were maintained in an Express Five SFM medium with 10% L-glutamine (ThermoFisher Scientific, Waltham, MA, USA) overnight. Then, 2 μg of the Myc-tagged *Lv*Perilipin expression plasmid or pIEX-4 vector (negative control) was transfected into cells using a FuGENE HD transfection reagent (Promega, Madison, WI, USA) according to the manufacturer’s instructions. The High Five cells were harvested at 36 h post-transfection and were lysed in a cell lysis buffer (20 mM Tris (pH 7.5), 150 mM NaCl, 1% Triton X-100) with the addition of 1 mM phenylmethylsulfonylfluoride (PMSF; BBI Life Sciences, Shanghai, China) for 30 min on ice. After that, the cell lysates were used for Western blotting analysis.

### 4.7. Western Blotting Analysis

The protein samples were separated by 10% sodium dodecyl sulfate-polyacrylamide gel electrophoresis (SDS-PAGE) and were transferred onto a PVDF transfer membrane (Millipore, Burlington, VT, USA). After blocking the membrane with 5% (*w*/*v*) skim milk dissolved in a TBST buffer (20 mM Tris, 150 mM NaCl, 0.1% Tween 20, pH 7.6) at room temperature for 1 h, the anti-Myc primary antibody (Cell Signaling Technology, Boston, MA, USA) was used to incubate the membrane for 1 h at room temperature. Then, the membranes were further incubated with a horseradish peroxidase (HRP)-conjugated secondary antibody (Thermo Fisher Scientific, Waltham, MA, USA) at room temperature for 1 h after washing three times with TBST. After that, Immobilon Western Chemiluminescent HRP Substrate (Millipore, Burlington, VT, USA) and Amersham Imager 600 (GE Healthcare, Chicago, IL, USA) were used for signal detection.

### 4.8. Immunofluorescence Analysis

The High Five cells were maintained in the Express Five SFM medium (Gibico, New York, USA) with 10% L-Glutamine (Gibico, USA) and were transfected with a pIEX-4 vector or Myc-tagged *Lv*Perilipin expression vector and were cultured for 36 h at 28 °C in 35 mm confocal Petri dishes (Cellvis, Sunnyvale, USA). After washing three times with PBS, 4% paraformaldehyde was used to fix the cells at room temperature for 15 min, and 0.5% TritonX-100 (diluted in PBS) was used to permeabilize the cells for 2 min. After washing, the cells were blocked with QuickBlock™ Blocking Buffer for Immunol Staining (Beyotime, Shanghai, China) for 1 h, followed by incubation overnight (about 10 h) at 4 °C with a mouse anti-Myc antibody (Cell Signaling Technology, USA; 1:300). Then, the cells were subsequently incubated with Alexa Fluor 488 goat anti-mouse (Beyotime, Shanghai, China) for 1 h at room temperature. After washing three times, Nile Red (ThermoFisher Scientific, Waltham, MA, USA) was used to stain the LDs in a final concentration of 0.1 μM for 10 min at room temperature. The cells were washed with PBS five times before the nuclei were stained with 1 × Hoechst 33342 (Beyotime, Shanghai, China) for 10 min. Finally, the cells were observed under a confocal laser-scanning microscope (Carl Zeiss LSM 800, CarlZeiss, Oberkochen, Germany).

### 4.9. Lipid Droplets Marking and Observation in Shrimp Hemocytes

Shrimp hemocytes were collected in 4% paraformaldehyde and anti-coagulant (27 mM trisodium citrate dihydrate, 33 mM citric acid, 110 mM glucose, 140 mM NaCl, pH 6.0) mixtures (1:1). The hemocytes were then washed three times with PBS and centrifuged at 500× *g* for 8 min to remove the plasm and mixtures. After re-suspending in an Insect-XPRESS medium (Lonza, Switzerland), the hemocytes were dropped onto 35 mm glass-bottom slides and were left to stand for 30 min. The cell slides were fixed with 0.5% TritonX-100 at room temperature for 1 min and were washed with PBS three times. The slides were stained with 0.1 μM Nile Red (ThermoFisher Scientific, USA) for 10 min in the dark, washed with PBS three times again, and then stained with Hoechst 33342 (Beyotime, Nanjing, China) for 10 min at room temperature. After washing four times with PBS for 5 min each, the slides were sealed with anti-fade solution (Beyotime, Nanjing, China) for confocal microscopic observation.

### 4.10. ROS Detection

In order to determine the role of *Lv*Perilipin in the production of total ROS during *Vp* (AHPND) infection in shrimp hemocytes, the ROS level was detected using the Reactive Oxygen Species Assay Kit (Beyotime, Shanghai, China) following the manufacturer’s instructions. In brief, hemocytes were collected after the RNAi assay, as described in Section 4.4, followed by incubating the cells with the 10 μM DCFH-DA fluorescence probe for 20 min at 28℃. After washing with a serum-free medium three times, the samples were analyzed using the Synergy H1 microplate reader (BioTek Instruments, Winooski, VT, USA) or the confocal laser-scanning microscope (Carl Zeiss LSM 800).

## Data Availability

All data and accession number(s) can be found in the article.

## Supplementary Materials

The supporting information can be downloaded at: https://www.mdpi.com/article/10.3390/ijms231810520/s1.
