# Supplementary material for: Shrimp Lipid Droplet Protein Perilipin Involves in the Pathogenesis of AHPND-Causing Vibrio parahaemolyticus
Source: Int J Mol Sci. 2022 Sep 10;23(18):10520. doi: 10.3390/ijms231810520 (PMC9501514; doi:10.3390/ijms231810520)
Supplement: Supplementary file 1 [file ijms-23-10520-s001.zip › Table S1.pdf]

Table S1 Primers used in this study

Primer name	Sequence (5'-3')
**For plasmid construction**	
Myc-*Lv*Perilipin-F	CGCGGATCCCGAGCAGAAACTCATCTCTGAAGAGGATCTGATGG CCCCCAAGCACCCCCACCTG
Myc-*Lv*Perilipin-R	CCCAAGCTTCTAATCTTTTGTCCTCCTCCTGATC
**For qRT-PCR assay**	
qRT-*Lv*Perilipin-F	CCTTCATCTTCGCCTCACA
qRT-*Lv*Perilipin-R	CACGCCCAGTTATCCACA
qRT-PirB-F	ACCAACAGCAGGTGAATA
qRT-PirB-R	GTGGGCTGATAACGACTC
qRT-*Lv*EF1α-F	TATGCTCCTTTTGGACGTTTTGC
qRT-*Lv*EF1α-R	CCTTTTCTGCGGCCTTGGTAG
**For RNAi assay**	
dsRNA-*Lv*Perilipin-F	GGGAGGCTGTGGATAACTG
dsRNA-*Lv*Perilipin-R	ATGGGCAGGGAAGCGACT
dsRNA-*Lv*Perilipin-T7-F	GGATCCTAATACGACTCACTATAGGGGGAGGCTGTGGATAACTG
dsRNA-*Lv*Perilipin-T7-R	GGATCCTAATACGACTCACTATAGGATGGGCAGGGAAGCGACT
dsRNA-EGFP-F	CGTAAACGGCCACAAGTT
dsRNA-EGFP-R	TTCACCTTGATGCCGTTC
dsRNA-EGFP-T7-F	GGATCCTAATACGACTCACTATAGGCGTAAACGGCCACAAGTT
dsRNA-EGPF-T7-R	GGATCCTAATACGACTCACTATAGGTTCACCTTGATGCCGTTC
